# Potentiation of tumour apoptosis by human growth hormone via glutathione production and decreased NF-*κ*B activity

**DOI:** 10.1038/sj.bjc.6601223

**Published:** 2003-09-09

**Authors:** C Cherbonnier, O Déas, G Carvalho, G Vassal, A Dürrbach, A Haeffner, B Charpentier, J Bénard, F Hirsch

**Affiliations:** 1 INSERM U542/Paris XI University, Villejuif Cedex, France; 2 Targa Therapies, Villejuif Cedex, France; 3 Institut Gustave Roussy and UMR8532, Villejuif Cedex, France; 4 Genetic Markers Unit, Institut Gustave Roussy, Villejuif, France

**Keywords:** growth hormone, apoptosis, glutathione, NF-*κ*B

## Abstract

In addition to its primary role as growth factor, human growth hormone (hGH) can also participate in cell survival, as already documented by its protective effect on human monocytes or human promyelocytic leukaemia U937 cells exposed to a Fas-mediated cell death signal. However, despite similarities in the molecular events following Fas and TNF-*α* receptor engagement, we report that U937 cells, genetically engineered to constitutively produce hGH, were made more sensitive to TNF-*α*-induced apoptosis than parental cells. This was due to overproduction of the antioxidant glutathione, which decreased the nuclear factor (NF)-*κ*B activity known to control the expression of survival genes. These findings were confirmed *in vivo*, in nude mice bearing U937 tumours coinjected with recombinant hGH and the NF-*κ*B -inducing anticancer drug daunorubicin, to avoid the *in vivo* toxicity of TNF-*α*. This study therefore highlights one of the various properties of hGH that may have potential clinical implications.

It has now been clearly established that the human growth hormone (hGH), secreted by the pituitary gland, exerts various important properties in addition to its fundamental role as a growth factor (reviewed in van Buul-Offers and Kooijman, 1998). This finding led to the use of hGH as adjuvant therapy in various diseases, such as AIDS (Lee, 1996), heart failure (Sacca and Fazio, 1996) or Crohn's disease (Slonim *et al*, 2000). Other recent studies have also established the effect of hGH on cell survival, as hGH may protect cells against various cell death inducers, such as irradiation (Mylonas *et al*, 2000) or growth factor deprivation, via nuclear factor (NF-*κ*B)-*κ*B activation (Jeay *et al*, 2000, 2001). Nuclear factor-B/Rel transcription factors have emerged as key factors in the regulation of inflammatory and immune responses as well as cell survival (Pahl, 1999). For instance, the injection of antisense to the p65 subunit of NF-*κ*B inhibited the growth of murine fibrosarcoma *in vivo* (Higgins *et al*, 1993) or murine and human squamous cell carcinomas, *in vitro* (Duffey *et al*, 1999; van Hogerlinden *et al*, 1999). Recently, Davis *et al*, (2001) comparing gene expression profiling genes in diffuse large B-cell lymphoma cells and in normal B cells from germinal centre clearly showed an overexpression of NF-*κ*B-targeted genes in tumour cells only, which may participate in their survival cells (Davis *et al*, 2001). Alternatively, inhibition of NF-*κ*B activation may result in an enhanced response to anticancer drug-induced tumour cell death. This has been clearly evidenced in various cancer cells by transient transfection of NF-*κ*B subunits (Liu *et al*, 1996) or stable transfection of gene encoding super-repressor form of I*κ*B, the natural inhibitor of NF-*κ*B (Baeuerle and Baltimore, 1988). In these situations, chemosensitivity was restored, as exemplified in drug-exposed human pancreatic carcinoma cells *in vitro* (Arlt *et al*, 2001), in murine fibrosarcoma *in vitro* (Van Antwerp *et al*, 1996; Wang *et al*, 1996) and *in vivo* (Wang *et al*, 1999a), or more recently in human colorectal cancer cells *in vivo* (Cusack *et al*, 2000). All these effects may be controlled via the NF-*κ*B-mediated transcription of survival genes, some of which have already been described, such as the inhibitor-of-apoptosis (IAP) (Wang *et al*, 1998) or immediate-early response gene *IEX-*1L (Wu *et al*, 1998), or suppression of proapoptotic cytochrome *c* release from mitochondria via activation of A1/Bfl-1, a member of the Bcl-2 family (Wang *et al*, 1999b). More recently, it has been proposed that NF-*κ*B may blunt the *c*-Jun amino-terminal kinase (JNK)-dependent cell death pathway by the transcription of JNK signalling inhibitors (De Smaele *et al*, 2001; Tang *et al*, 2001). The NF-*κ*B pathway may, therefore, be a suitable target for anticancer therapy.

In a recent study, we also reported that hGH may modulate the response of cells to apoptotic signals, demonstrating its beneficial effect on the survival of human monocytes and promyelocytic leukaemia U937 cells exposed to a proapoptotic signal mediated via Fas engagement (Haeffner *et al*, 1999). In parallel, we exposed our U937 cell lines to TNF-*α*, expecting similar protection, as Fas is a member of the tumour necvosis factor (TNF) receptor superfamily (Itoh *et al*, 1991; Oehm *et al*, 1992), and shares common signalling pathways with the type 1 TNF receptor (Hsu *et al*, 1996), including in U937 cells (Schütze *et al*, 1992; Cifone *et al*, 1993). In contrast, as reported herein, we observed that hGH sensitised U937 cells to the apoptotic signal induced by TNF-*α*. The effect of hGH is mediated via a glutathione-dependent decrease in NF-*κ*B translocation normally observed in response to TNF-α. We also confirmed these findings *in vivo*, as injections of exogenous recombinant hGH in U937 tumour-engrafted nude mice led to increased tumour cell death together with decreased translocation of NF-*κ*B in cell nuclei, in response to subtoxic doses of daunorubicin, an anthracycline agent activating NF-*κ*B in our cells (Cherbonnier *et al*, 2002).

## MATERIALS AND METHODS

### Cells and culture conditions

Two different human myeloid leukaemia U937 cell lines producing 10–50 ng ml^−1^ of hGH after stable gene transfer (U937-hGH-A and U937-hGH-H5) and the control line (U937-Neo), already described (Haeffner *et al*, 1999), were maintained in regular FCS-enriched RPMI 1640 medium (Bio-Whittaker Europe, Verviers, Belgium) containing 0.5 mg ml^−1^ of G418 (Life Technology, Paisley, Scotland).

### Assessment of apoptosis

U937 cells (5 × 10^5^) (the two hGH-transfected and the control lines) in 0.5 ml of culture medium were cultured for 48 h in the presence or absence of TNF-*α* (Innotest, Besançon, France) with or without 400 ng ml^−1^ of cycloheximide (CHX, Sigma, St Louis, MO, USA), as previously reported (Cossarizza *et al*, 1995). Dead cells were detected either by trypan blue exclusion for experiments run in the presence of recombinant hGH (rhGH, Saizen®, kindly provided by Serono France) at doses already experimented (Haeffner *et al*, 1999) or by flow cytometry analysis of cell lines after a 15 min staining with propidium iodide (PI) (5 *μ*g ml^−1^ pi, Sigma, St-Louis, MO, USA), using a FACScan (Becton Dickinson, Mountain View, CA, USA). Hypoploid cell assessment was measured on ethanol-permeabilised U937 cells exposed or not to 10 ng ml^−1^ of TNF-*α* cultured overnight with or without 400 ng ml^−1^ of cycloheximide (CHX), by staining with PI, as previously described (Mollereau *et al*, 1996). In separate experiments, we added glutathione ethyl ester (GSH-OEt) (Sigma) to the culture of control U937 cells in the presence or absence of TNF-*α*.

### NF-*κ*B measurement

Cell lines were cultured for 90 min in the presence of 10 ng ml^−1^ TNF-*α*, and nuclear extracts were analysed by electrophoretic mobility-shift assay (EMSA). Specificity was assessed by incubating nuclear extracts obtained from TNF-*α*-stimulated cells with nonradiolabelled NF-*κ*B probe (5′-ACAAGGGACTTTCCGCTGGGGACTTTCCAG-3′) or mutated NF-B (5′-ACAACTCACTTTCCGCTGCTCACTTTCCAG-3′) oligonucleotide probe, as previously reported (Thieblemont *et al*, 1995). For supershift assays, anti-P50 and anti-P65 Abs (Santa Cruz Biotechnology, Santa Cruz, CA, USA) were used, as reported in U937 cells (Giri *et al*, 1998). In separate experiments, we analysed nuclear extracts from U937 cell lines cultured in the absence or in the presence of TNF-*α* (10 ng ml^−1^, 90 min)±rhGH. In other separate experiments, we analysed nuclear extracts from U937 cell lines cultured in the absence or in the presence of: TNF-*α* (10 ng ml^−1^, 90 min)±CHX (400 ng ml^−1^, 90 min), or anti-Fas mAb (CH-11 anti-Fas mAb, Immunotech, Marseille, France) (1 *μ*g ml^−1^, 90 min). In both cases, specificity was assessed by using the probes described above. Alternatively, prior to nuclear extractions, U937 cells were incubated with 10 ng ml^−1^ of TNF-*α* alone or in addition to various doses of glutathione ethyl-ester (GSH-OEt, Sigma).

### I*κ*B measurement

Cell lysates obtained from 5 × 10^5^ cells with or without exposure to 5 ng ml^−1^ of TNF-*α* were separated on 10% SDS–polyacrylamide gel and electroblotted onto nitrocellulose membranes. In separate experiments, U937 cells were incubated with 5 ng ml^−1^ of TNF-*α* alone or in addition to 20 mM of GSH and U937-hGH cells were incubated with 5 ng ml^−1^ TNF-*α* alone or in addition to 50 mM of diethyl maleate (DEM) (Sigma), in conditions already used in our models (Déas *et al*, 1997). The same amounts of proteins based on Bradford measurement were loaded onto the gel. Blots were first stained with rabbit anti-I*κ*B*α* (Santa-Cruz Biotechnology) or anti-I*κ*B Abs (kindly provided by A Israël, Institut Pasteur) after striping, and then with anti-rabbit peroxidase-labelled second Abs (Amersham). They were developed using an enhanced chemiluminescence detection system (ECL, Amersham, UK).

### Glutathione measurement

The total glutathione level was measured by the glutathione reductase recycling method, as previously described (Déas *et al*, 1997). Glutathione is expressed in nmol mg^−1^ of total protein measured in cell extracts by the Bradford method.

### *In vivo* experiments

U937 cells (10^7^) were first injected subcutaneously into irradiated 6- to 8-week-old male nu/nu Swiss mice in order to obtain solid tumours. Another group of mice was then engrafted with fragments from these tumours (4 mm^3^), pooled and randomly assigned to four groups of 10 mice: animals injected with saline solution; animals injected with 1.5 mg kg^−1^ of daunorubicin (dosage determined as previously reported (Cherbonnier *et al*, 2002); animals injected with 5 mg kg^−1^ of recombinant exogenous hGH (rhGH) based on the study of the *in vivo* effects of rhGH in a murine model of sepsis induced by *Escherichia coli* (Inoue *et al*, 1995); and animals injected with 1.5 mg kg^−1^ of daunorubicin and 5 mg kg^−1^ of rhGH. Daunorubicin was administered as a single intraperitoneal injection on three consecutive days. Recombinant exogenous rhGH was administered subcutaneously as a single injection on four consecutive days, starting 2 days prior to the first injection of daunorubicin. Animal body weights were recorded and tumours were measured with calipers by the same investigator and the volume was calculated according to the following equation: *V* (mm^3^)=*d*^2^ (mm^2^) × *D* (mm)/2, where *d* and *D* are the smallest and perpendicular diameters, respectively. For ethical reasons, the maximum tumour volume accepted was 2000 mm^3^. In a separate experiment, mice were treated as described above and were killed at various times. Fixed tumours were analysed by epifluorescence for *in situ* apoptosis with terminal-deoxynucleotidyl-transferase-mediated dUTP nick-end labelling (TUNEL) (Roche) or for *in situ* NF-*κ*B activation with rabbit anti-P65 Abs revealed with fluorescein-labelled goat anti-rabbit Abs, and nuclei were stained with DAPI. Animals were killed under CO_2_ anaesthesia. All experiments were carried out under the conditions established by the European Union (Directive 86/609/EEC).

### Statistical analysis

Statistical analyses were performed using Student's *t*-test for *in vitro* experiments. *In vivo* experiments were assessed using a nonparametric Kruskal–Wallis test for tumour volume measurement, using logrank test for Kaplan–Meier curves and a χ^2^ test for the quantification of apoptosis and NF-*κ*B activation on tissue sections.

## RESULTS

### Human growth hormone increases the sensitivity to TNF-*α*-induced apoptosis

**Figure 1:**
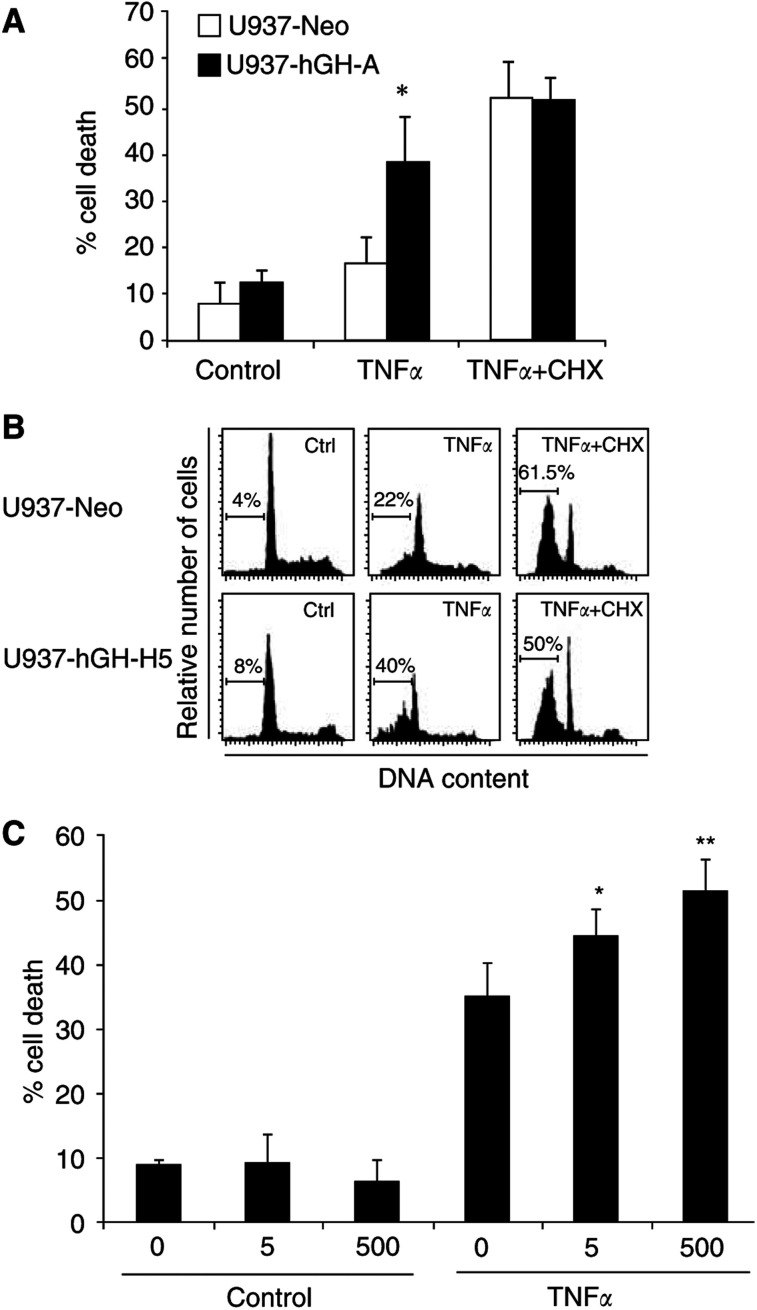
Effect of hGH on TNF-*α*-induced apoptosis. U937-Neo and U937-hGH cell lines were cultured in the presence or absence of TNF-*α* (10 ng ml^−1^) with or without CHX (400 ng ml^−1^) (**A**) The percentage of dead cells was measured by PI staining. The values represent the mean±s.e.m. of six independent experiments (^*^P=0.001). (**B**) The percentage of cells with hypodiploid DNA content was detected by PI staining of ethanol-permeabilised cells. Linear scales are represented. (**C**) U937 cells were cultured for 48 h in the presence or absence of 50 ng ml^−1^ TNF-*α* with or without exogenous recombinant hGH at 5 or 500 ng ml^−1^. The percentage of dead cells was measured by trypan blue exclusion. The values represent the mean±s.e.m. of four experiments (^*^P<0.05, ^**^P<0.01).

Based on our previous studies demonstrating that stable expression of hGH in cell lines could mimic the effect of exogenous GH (Haeffner *et al*, 1999), we exposed our two hGH-producing human myeloid leukaemia U937 cell lines and the control one to TNF-*α* during 2-day cultures. As illustrated in Figure 1A

, the percentage of dead cells (PI+) was higher for U937-hGH cells than for control U937-Neo cells, in response to TNF-*α* (37.5±9.6% of PI+U937-hGH cells treated with 10 ng ml^−1^ of TNF-*α*, *vs* 16.7±5.6% of PI+cells among TNF-*α*-treated control cell lines, mean±s.e.m., *P*=0.001, *n*=6). In contrast, hGH had no additional effect on apoptosis of cells exposed to TNF-*α* in the presence of the protein synthesis inhibitor CHX, known to potentiate TNF-*α*-induced U937 cell death (Cossarizza *et al*, 1995). The difference between hGH-producing cells and their controls was also demonstrated in terms of apoptotic nuclear events, as evidenced by flow cytometry of permeabilised cells allowing DNA content analysis (Figure 1B). Under these conditions, after 20-h cultures, presence of hGH led also to an increase in hypodiploid DNA in TNF-*α*-treated cells. Finally, we confirmed that exogenous rhGH added to the cultures of unmanipulated U937 cells rendered them more sensitive to TNF-*α*-treatment (Figure 1C). Indeed, in these conditions, we observed a statistically significant increased cell death in hGH-treated U937 cells stimulated with 50 ng ml^−1^ of TNF-*α*, as compared to U937 cells cultured in the absence of rhGH (44.6±0.7% of dead U937 cells treated with 50 ng ml^−1^ of TNF-*α* in the presence of 5 ng ml^−1^ of rhGH and 51.5±5.5% of dead U937 cells treated with 50 ng ml^−1^ of TNF-*α* in the presence of 500 ng ml^−1^ of rhGH, *vs* 35.2±0.6% among cells treated with TNF-*α*- in the absence of rhGH, mean±s.e.m., *P*<0.05 and *P*<0.01, respectively, *n*=4).

### Specific depressed NF-*κ*activation in cells treated with TNF-*α* by hGH

**Figure 2:**
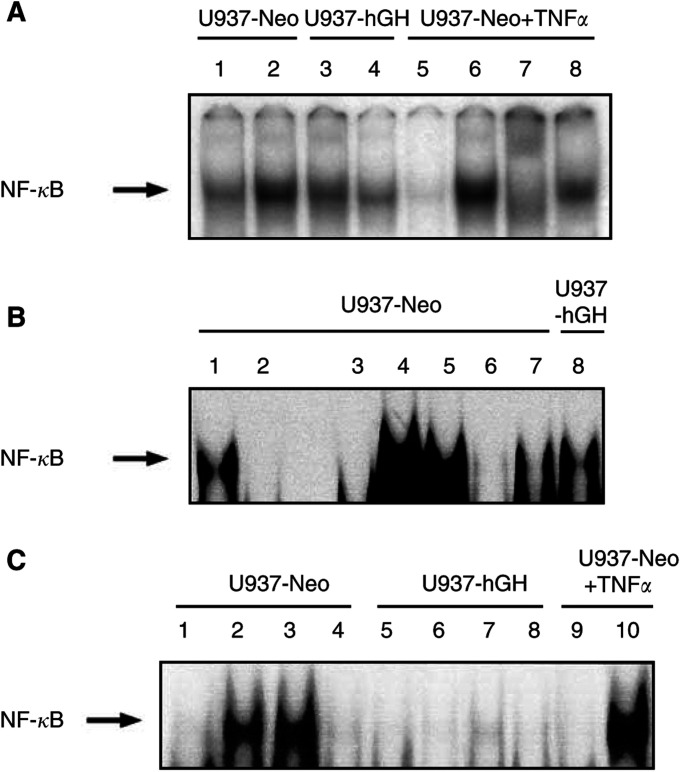
Effect of hGH on NF-*κ*B activation. (**A**) An EMSA was performed on nuclear extracts as described in Materials and Methods. Control U937 or *hGH*-transfected cells were either nonstimulated (lanes 1 and 3, respectively) or stimulated with TNF-*α* (10 ng ml^−1^) (lanes 2 and 4, respectively). Nuclear factor-*κ*B migration was assessed by migration of nuclear extracts from U937-Neo cells stimulated with TNF-*α*, coincubated with nonlabelled NF-*κ*B-specific (lane 5) or NF-*κ*B-mutated probes (lane 6), or with anti-P65 (lane 7) and anti-P50 (lane 8) NF-*κ*B subunit antibodies. (**B**) In a separate experiment, an EMSA was performed on nuclear extracts from U937-Neo cells cultured in the absence or in the presence of TNF-*α* (10 ng ml^−1^) (lanes 3 and 4, respectively), coincubated with 5, 50, or 500 ng ml^−1^ of rhGH (lanes 5, 6 and 7, respectively). U937-hGH cells were also stimulated with TNF-*α* (10 ng ml^−1^) (lane 8). Nuclear factor-*κ*B migration was assessed by migration of nuclear extracts from U937-Neo cells stimulated with TNF-*α*, and coincubated with nonlabelled NF-*κ*B-specific (lane 2) or NF-*κ*B-mutated (lane 1) probes. (**C**) In another experiment, U937-Neo and U937-hGH cells were either nonstimulated (lanes 1 and 5, respectively), stimulated with TNF-*α* (10 ng ml^−1^) (lanes 2 and 6, respectively), stimulated with TNF-*α* and CHX (400 ng ml^−1^) (lanes 3 and 7, respectively) or stimulated with anti-Fas mAbs (lanes 4 and 8, respectively). Specificity was assessed by migration of nuclear extracts from U937-Neo cells stimulated with TNF-*α*, and coincubated with the probes described above, nonlabelled NF-*κ*B-specific (lane 9) or NF-*κ*B-mutated (lane 10) probes.

As inhibition of NF-*κ*B activation was clearly linked to increased apoptosis of various tumour cells, we decided to test NF-*κ*B DNA binding activity in nuclear extracts from our cell lines incubated with TNF-*α*, a potent NF-*κ*B stimulator (Baeuerle and Baltimore, 1988). As illustrated in Figure 2A

, the increase in NF-*κ*B-DNA binding activity observed in U937-Neo cells exposed to TNF-*α* (lane 2), as compared to nonexposed cells (lane 1), was no longer observed in U937-hGH cells treated under similar conditions (lanes 4 and 3, respectively). Specificity was assessed by incubating nuclear extracts obtained from TNF-*α*-treated control cells with nonradiolabelled consensus NF-*κ*B (lane 5) or mutated NF*κ*B (lane 6) probe. Interactions of these nuclear extracts were also observed with anti-P65 (lane 7) and, to a lesser extent, anti-P50 (lane 8) Abs, confirming the detection of NF-*κ*B.

To confirm the effect of hGH on NF-*κ*B activity, in separate experiment, parental U937 cells were coincubated with TNF-*α* and various concentrations of rhGH. As shown in Figure 2B, TNF-*α*-mediated NF-*κ*B-DNA binding activity (lane 4) was decreased by addition of 50 ng ml^−1^ (lane 6) or 500 ng ml^−1^ (lane 7) of rhGH.

NF-*κ*B can interact with Fas-mediated apoptosis in various situations (Zong *et al*, 1998; Dudley *et al*, 1999; Trauzold *et al*, 2001; Zheng *et al*, 2001), which prompted us to investigate the effect of hGH on NF-*κ*B translocation using nuclear extracts from cells exposed to anti-Fas mAb (Figure 2C). No protein–DNA complex was seen using extracts from U937-Neo and U937-hGH cells exposed to anti-Fas mAb (lanes 4 and 8, respectively). As expected, protein–DNA complexes were observed using nuclear extracts from U937-Neo cells exposed to TNF-*α* (lane 2), and also to TNF-*α*±CHX (lane 3), compared to unstimulated cells (lane 1). These protein–DNA complexes were specifically competed by a consensus NF-*κ*B probe but not by the mutated probe (lanes 9 and 10, respectively). The present experiment also confirmed that NF-*κ*B translocation was strongly inhibited by hGH in response to a TNF-*α* stimulus (lane 6), and was markedly diminished in response to a TNF-*α*+CHX stimulus (lane 7).

### Inhibition of TNF-*α* induced I*κ*B degradation by hGH

**Figure 3:**
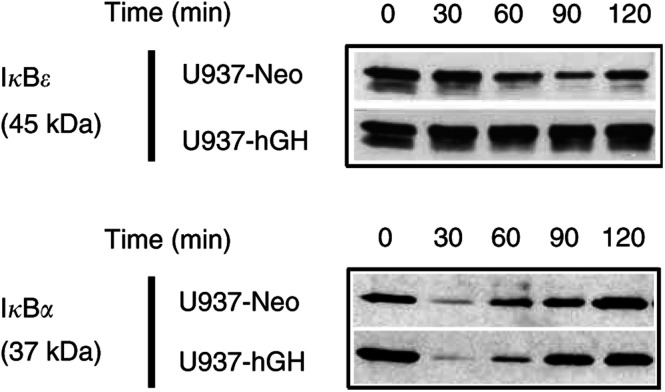
Effect of hGH on I*κ*B degradation. Whole-cell extracts from the indicated cell lines stimulated with TNF-*α* (10 ng ml^−1^) for 0–120 min were subjected to Western blotting using anti-I*κ*B Abs under the conditions described in Materials and Methods.

Intracellular localisation of NF-*κ*B can be closely monitored by its interaction with members of the I*κ*B family. In response to various stimuli, I*κ*B undergoes proteolytic degradation, which allows nuclear translocation of NF-*κ*B leading to gene activation (Baeuerle and Baltimore, 1988 #733). To determine whether the decreased activation of NF-*κ*B observed in hGH-treated cells was due to a modulation of I*κ* degradation, we performed Western blot analyses on whole-cell extracts from our cell lines. After TNF-*α* exposure, degradation of *κ*B was observed in U937-Neo cells, while no degradation was detected in U937-hGH cells, even after incubation for 2 h (Figure 3

). Interestingly, I*κ*B*α* (Figure 3) and IκBβ (not shown) were degraded to a similar degree in U937-Neo control and in hGH-producing U937-Neo-cells exposed to TNF-*α*.

### Effects of hGH-induced enhanced GSH production

**Figure 4:**
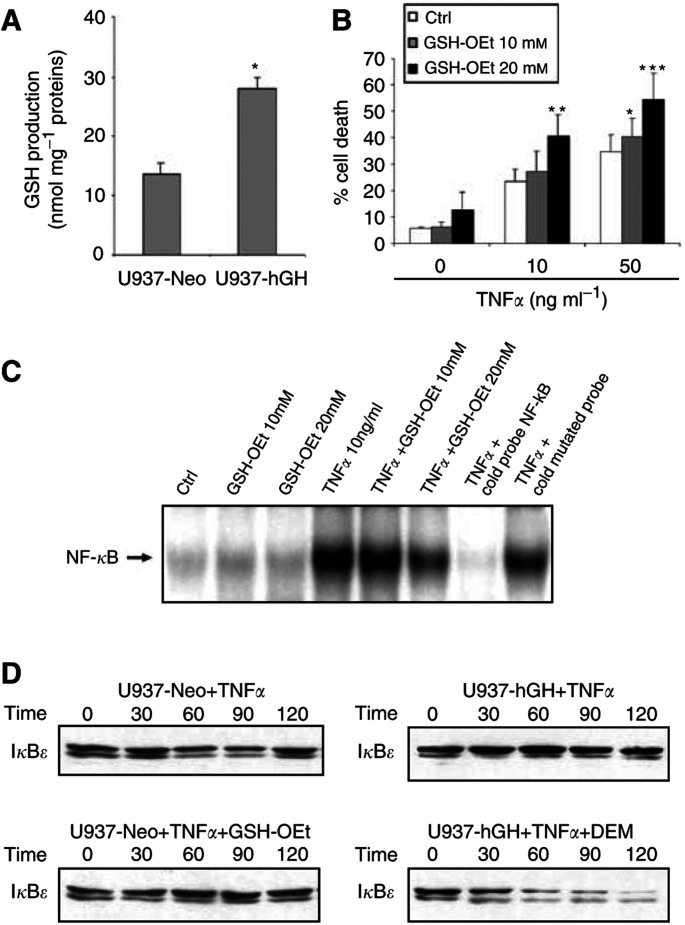
(A) Effect of hGH on GSH production. The glutathione level was measured in U937-Neo and U937-hGH cells under the conditions described in Materials and Methods. The values represent the mean±s.e.m. of four independent experiments (^*^P<0.05). (**B**) Effect of GSH-OEt on TNF-*α*-induced apoptosis. U937-Neo cells were stimulated with TNF-*α* in the presence or absence of GSH-OEt and under the conditions described in Materials and Methods: the percentage of dead cells was measured by PI staining. The values represent the mean±s.e.m. of three independent experiments (^*^P<0.04, ^**^P<0.03, ^***^P<0.02), when comparing GSH-OEt treated cells to GSH-OEt non-treated cells in each group. (**C**) Effect of GSH-OEt on NF-*κ*B activation. Nuclear extracts were analysed by EMSA for measurement of NF-*κ*B activation, as described in Figure 2. (**D**) Effect of GSH on I*κ*B degradation. Whole-cell extracts were subjected to Western blotting using anti-I*κ*B Abs, as described in Figure 3.

The activation of NF-*κ*B in response to various agents requires the production of reactive oxidative intermediates (ROIs) (Janssen-Heininger *et al*, 2000). Since glutathione, the most significant intracellular thiol, is a potent ROI scavenger (Meister and Anderson, 1983) with the potential to prevent NF-*κ*B activation (Janssen-Heininger *et al*, 2000), we first measured its production in our cells. As illustrated in Figure 4A

, U937-hGH cell lines produced more total intracellular GSH than control cells. We therefore decided to test whether direct addition of exogenous GSH to U937 control cells could mimic hGH treatment *in vitro*. Addition of glutathione ethyl ester (GSH-OEt), a GSH analogue which can enter cells due to its hydrophobic ester groups, increased cell death in TNF-*α*-treated U937 cells (Figure 4B). These results were statistically significant at 10 mM GSH-OEt for the highest TNF-*α* concentration used in this study, and at 20 mM GSH-OEt for both TNF-*α* concentrations. We then studied the effect of GSH-OEt on both NF-*κ*B activation and I*κ*B degradation. The presence of GSH-OEt depressed NF-*κ*B-DNA binding activity in a dose-dependent manner in U937-Neo cells treated with 10 ng ml^−1^ of TNF-*α* (Figure 4C). The presence of GSH-OEt (Figure 4D, lower left) in the culture prevented the degradation of I*κ*B that normally occurred upon TNF-*α*-treatment only (Figure 4D, upper left). Moreover, addition of DEM, a potent thiol-depleting agent, in cell cultures led to I*κ*B degradation in TNF-*α*-treated hGH-producing cells (Figure 4D, lower right), while, as expected, a pattern of stabilised I*κ*B was shown in U937-hGH cells with an unmodified thiol level (Figure 4D, upper right). Altogether, these results suggest that hGH may control the response to TNF-*α* by modifying the redox status of the cells.

### *In vivo* sensitisation of tumours by rhGH

**Figure 5:**
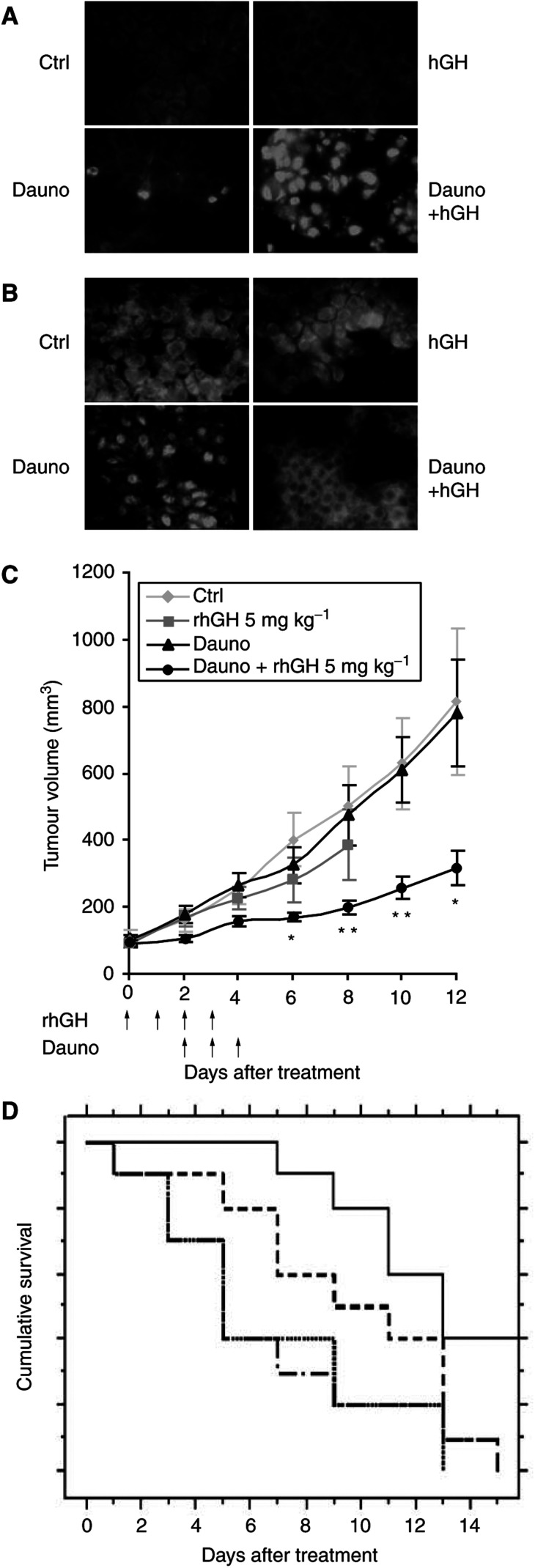
*In vivo* effect of hGH on daunorubicin treatment of engrafted tumours. Small tumour fragments obtained from U937-Neo tumours were transplanted subcutaneously in previously irradiated nude Swiss mice. Tumour-bearing mice either received i.p. injections of saline solution or subcutaneous injections of rhGH alone, or the highest subtoxic dose of daunorubicin (1.5 mg kg^−1^) with or without 5 mg kg^−1^ of rhGH. (**A**) Tissue sections from mice treated as above were submitted to TUNEL assay. Note the significant number of stained nuclei in tumours from rhGH- and daunorubicin-treated animals, indicating a high apoptosis rate in these animals. (**B**) The same tumours were assessed for NF-*κ*B activation, as evidenced by the presence of the P65 subunit in the nuclei. Note the lower number of stained nuclei in tumours from mice treated with rhGH and daunorubicin, compared with daunorubicin-treated animals. (**C**) Tumour volumes were measured in each group of mice. The measurement was stopped when a mouse died or was killed because of a tumour volume exceeding 2000 mm^3^. Each line represents the mean±s.e.m. of the tumour volumes (^*^P<0.05 and ^**^P<0.01, when comparing daunorubicin-treated mice to mice receiving daunorubicin and rhGH). (**D**) Kaplan-Meier curve comparing the different groups of mice with a tumour volume below 300 mm^3^. PBS-injected mice (….), daunomycine-injected mice (.-.-.-.-.) hGH-injected mice (- - -) and daunomycine+hGH-injected mice (---).

To extend and validate this study *in vivo*, we decided to use the anticancer drug daunorubicin, as TNF-*α*, one of the main cytokines involved in sepsis (reviewed in Dinarello, 2000), may induce devastating effects after *in vivo* injection. Moreover, daunorubicin, widely used in clinical practice, is able to induce apoptosis and activate NF-*κ*B in our cells (Cherbonnier *et al*, 2002). We therefore decided to study the effect of exogenous recombinant hGH on chemotherapy delivered to nude mice engrafted with parental unmodified U937 tumours. In order to verify the proposed mechanism explaining the effect of hGH, *in vivo*, we first examined *in situ* apoptosis in the various groups of mice defined in Materials and Methods. A TUNEL assay reported in Figure 5A

clearly showed a greater number of fluorescent nuclei, reflecting increased cell death in tumour cells of mice treated with both daunorubicin and rhGH compared to daunorubicin-treated mice (*P*<0.001), while no fluorescent nuclei were observed in the two other groups. In addition, the limited nuclear staining revealed with an anti-P65 NF-*κ*B subunit Ab, evidenced a marked decrease in NF-*κ*B activation in tumour cells of mice treated with both daunorubicin and rhGH, compared to the daunorubicin-treated group (*P*<0.0001) (Figure 5B). Both effects were observed 2 days after the last injection of daunorubicin (day 6). Similar analyses performed on days 9 and 12 showed equivalent number of dead cells and lack of NF-*κ*B in cell nuclei, in both daunorubicin-treated groups.

In parallel, the growth rate of U937 tumours was measured in these mice. As shown in Figure 5C, this rate was reduced in mice treated with the highest subtoxic dose of daunorubicin (1.5 mg kg^−1^, as defined in Materials and Methods) combined with injections of 5 mg kg^−1^ of rhGH, while tumours remained unaffected by treatment with daunorubicin only. Tumour volumes were significantly different from the 6th day after initiation of treatment until the 12th day, when comparing animals treated with rhGH and daunorubicin to animals treated with daunorubicin only (at day 6: 171±14 mm^3^ *vs* 327±53 mm^3^, *n*=10, ^*^P<0.05 and at day 12: 320±52 mm^3^ *vs* 781±159 mm^3^, *n*=10, ^*^P<0.05). No further comparisons were possible due to death of animals in the daunorubicin-treated group at day 12. No protective effects were observed in mice receiving saline (Ctrl) or rhGH only. Given the high growth rate of U937 cells *in vivo* and the design of the present experiments in which only a single cure was given to the mice, the complete eradication of tumours could not be achieved. However, the survival of mice with a tumour below 300 mm^3^ was determined. As shown in Figure 5D, the survival of mice treated with both daunorubicine and rhGH was significantly higher than in the different control groups (*P*<0.008).

It should be noted that coadministration of rhGH and daunorubicin did not induce any toxicity or body weight loss (data not shown).

## DISCUSSION

In a previous study, we demonstrated the ability of hGH to protect human monocytes or U937 cells from cell death induced by Fas engagement (Haeffner *et al*, 1999). Interestingly, despite molecular similarities between Fas and TNF-*α* receptors (Itoh *et al*, 1991) and common pathways in response to their engagement in various cell lines, including U937 cells (Schütze *et al*, 1992; Cifone *et al*, 1993), we report herein that hGH may potentiate TNF-*α*-induced U937 cell apoptosis (Figure 1). It has been clearly established that the cell death signal mediated via engagement of TNF-*α* receptor can be regulated by NF-*κ*B activation subunits (Liu *et al*, 1996); (Van Antwerp *et al*, 1996; Wang *et al*, 1996). In our study, we found that NF-*κ*B activation was markedly decreased in U937-hGH cells exposed to TNF-*α* compared to treated U937-Neo cells (Figure 2A) and in parental cells incubated with exogenous rhGH (Figure 2B). These results therefore confirm our previous findings (Haeffner *et al*, 1997) concerning the ability of hGH to modulate the NF-*κ*B-dependent pathway in response to various inducers. Fas crosslinking may induce death or survival signalling, depending on the cell type (Budd, 2002). Survival is linked to the engagement of FLICE inhibitory protein (FLIP) that may phosphorylate I*κ*B, thereby activating NF-*κ*B. As anti-Fas mAbs were unable to induce NF-*κ*B activation in our cells (Figure 2C), hGH may trigger another pathway promoting cell survival in response to Fas. This difference in NF-*κ*B profile could therefore partly explain the opposite effects observed with Fas and TNF-*α*.

As also reported in Figure 1, addition of protein synthesis inhibitor CHX, in control cells cultured with TNF-*α*, induced an expected increase in cell apoptosis (Cossarizza *et al*, 1995). Under similar conditions, hGH, although still synthesised, as assessed by ELISA (data not shown) was unable to improve cell death. Moreover, the presence of CHX did not modify the NF-*κ*B profile in either U937-hGH or U937-Neo cells exposed to TNF-*α* (Figure 2C). We can therefore speculate that hGH and CHX both increase apoptosis by affecting the synthesis of survival proteins: hGH acts upstream to NF-*κ*B activation, while CHX acts downstream to NF-*κ*B activation.

Activation of NF-*κ*B, that is, its translocation in the cell nucleus, occurs after proteosomal degradation of members of the I*κ*B family following phosphorylation by I*κ*B kinases (IKK) (Mercurio and Manning, 1999). One of these kinases, IKK*β* is essential for the prevention of TNF-*α*-induced apoptosis (Senftleben *et al*, 2001). The I*κ*B family is composed of several *α*, *β* and *ɛ* proteins constitutively expressed and present in the cytosol, all sharing the capacity to prevent NF-*κ*B translocation, and the newly described *ζ* protein localised in the nucleus (Yamazaki *et al*, 2001). Unexpectedly, we report that hGH regulated TNF-*α*-dependent NF-*κ*B activation via stabilisation of I*κ*B*ɛ*, and not I*κ*B*α*, the main member of the I*κ*B family (Figure 3), or I*κ*B*β* (data not shown). Although unusual, this type of situation has already been described by other authors in cells from I*κ*B *α*-deficient mice in response to TNF-*α* (Whiteside *et al*, 1997), or in other models (Doerre and Corley, 1999; Spiecker *et al*, 2000). Fischer *et al* (1999) also reported that TNF-*α* may activate NF-*κ*B via degradation of I*κ*B*ɛ* in human monocytic THP1 cells. TNF-*α*-inducing NF-*κ*B activation may therefore involve various members of the I*κ*B family, probably depending on the cell type.

Tumour necrosis factor-*α* induces the release of ROIs (Goossens *et al*, 1995) that are also considered to be potent NF-*κ*B activators in many cell types, including tumour cells (Giri *et al*, 1998; Li and Karin, 1999; Manna *et al*, 2000). We therefore postulated that hGH may act on apoptosis by downmodulation of ROI production. In order to evaluate this hypothesis, we first assessed the effect of hGH on the level of GSH, the most abundant intracellular thiol and a powerful ROI scavenger. We found that hGH increased GSH production in our tumour cells (Figure 4A). In parallel, we observed that addition of GSH-OEt to unmodified U937 cells exposed to TNF-*α* led to inhibition of NF-*κ*B activation by I*κ*B*ɛ* stabilisation (Figure 4C and D), thus mimicking the effect of hGH. Moreover, GSH depletion with 50 mM DEM, a potent thiol-depleting agent, in hGH-producing cells exposed to TNF-*α* restored the I*κ*B degradation process (Figure 4D). Direct addition of a GSH analog (GSH-OEt) able to enter cells due to its hydrophobic ester groups also increased cell death in TNF-*α*-treated cell cultures (Figure 4B), thus imitating the ability of hGH to sensitise control U937 cells to TNF-*α*-induced apoptosis. These last data are in apparent contradiction with several studies, including our own, run in other cell types, showing that GSH may protect cells from a death signal (Déas *et al*, 1997). However, in line with our findings, a report of experiments in U937 cells clearly revealed that antioxidant reagents may block NF-*κ*B activation and potentiate TNF-*α*-induced apoptosis (Shrivastava and Aggarwal, 1999). Altogether, our findings clearly suggest that hGH controls NF-*κ*B activation and therefore apoptosis via modification of the redox status of tumours exposed to the anticancer drugs used in this study. Thus, depending on the stimulus applied to the cells, hGH may either trigger (Jeay *et al*, 2000), inhibit (as we have reported in human monocytes exposed to lipopolysaccharides (Haeffner *et al*, 1997) or decrease (as in the present study) translocation of NF-*κ*B into cell nuclei.

We finally decided to validate our findings in an *in vivo* model. Owing to the high toxicity of TNF-*α in vivo*, we decided to treat mice with the anticancer drug daunorubicin, one of the most commonly used and broadly active chemotherapeutic agents. Moreover, daunorubicin shares several properties with TNF-*α*, that is, NF-*κ*B stimulation (Das and White, 1997) and ROI production (Gewirtz, 1999). This latter effect was correlated to deleterious effects, such as severe cardiomyopathy observed in cancer patients (Gewirtz, 1999). We then investigated the effect of recombinant hGH injections in nude mice bearing xenografted tumours concomitantly treated with the highest dose of daunorubicin previously demonstrated to be devoid of animal toxicity. The efficacy of hGH was confirmed by these experiments as, 6 days after initiation of treatment, greater numbers of apoptotic cells (Figure 5A) and cells displaying NF-*κ*B inhibition (Figure 5B) were observed in mice treated with both drug and rhGH than in the other groups of mice. In parallel, the U937 tumour cell growth rate was significantly decreased in mice treated with both daunorubicin and rhGH, compared to U937 tumours in mice receiving daunorubicin or rhGH or saline alone (Figure 5C). We speculate that more prolonged treatment would extend and improve the efficacy of the therapeutic outcome.

Finally, our model may help to shed some light on one of the various mechanisms controlling NF-*κ*B activation. Following binding to its receptors, hGH induces activation of the Janus kinase 2 (Jak2) (Argetsinger *et al*, 1996), which in turn tyrosine-phosphorylates signal transducers and activators of transcription proteins (STAT) (Ram and Waxman, 1997). These phosphorylated proteins migrate into the nucleus and, by binding to GH-responsive elements, trigger gene transcription. These data must be interpreted in the light of the recently proposed crosstalk between NF-*κ*B and STAT showing either an NF-*κ*B-mediated negative control of STAT phosphorylation (Geymayer and Doppler, 2000), or alternatively, the inhibition of NF-*κ*B mediated by STAT phosphorylation (Luo and Yu-Lee, 2000). It is therefore conceivable that hGH may regulate NF-*κ*B and consequently cell apoptosis via a Jak-STAT-dependent mechanism; this hypothesis is currently being investigated. This should be of interest in the development of new therapeutic modalities designed to improve anticancer chemotherapy.
